# Multi-omics analyses based on genes associated with oxidative stress and phospholipid metabolism revealed the intrinsic molecular characteristics of pancreatic cancer

**DOI:** 10.1038/s41598-023-40560-4

**Published:** 2023-08-21

**Authors:** Hongdong Wang, Hui Guo, Jiaao Sun, Yuefeng Wang

**Affiliations:** 1 Department of Hepatobiliary Pancreatic Surgery, The Second Affiliated Hospital of Dalian Medical University, Dalian, China; 2 Department of General Surgery, The First Affiliated Hospital of Dalian Medical University, Dalian, China; 3 Department of Urology, The First Affiliated Hospital of Dalian Medical University, Dalian, China

**Keywords:** Cancer, Computational biology and bioinformatics

## Abstract

Oxidative stress (OS), which impacts lipid metabolic reprogramming, can affect the biological activities of cancer cells. How oxidative stress and phospholipid metabolism (OSPM) influence the prognosis of pancreatic cancer (PC) needs to be elucidated. The metabolic data of 35 pancreatic tumor samples, 34 para-carcinoma samples, and 31 normal pancreatic tissues were obtained from the previously published literature. Pan-cancer samples were obtained from The Cancer Genome Atlas (TCGA). And the Gene Expression Omnibus (GEO), International Cancer Genome Consortium (ICGC), ArrayExpress, and the Genotype-Tissue Expression (GTEx) databases were searched for more PC and normal pancreatic samples. The metabolites in PC were compared with normal and para-carcinoma tissues. The characteristics of the key OSPM genes were summarized in pan-cancer. The random survival forest analysis and multivariate Cox regression analysis were utilized to construct an OSPM-related signature. Based on this signature, PC samples were divided into high- and low-risk subgroups. The dysregulations of the tumor immune microenvironment were further investigated. Quantitative reverse transcription polymerase chain reaction (qRT-PCR) was conducted to investigate the expression of genes in the signature in PC and normal tissues. The protein levels of these genes were further demonstrated. In PC, metabolomic studies revealed the alteration of PM, while transcriptomic studies showed different expressions of OSPM-related genes. Then 930 PC samples were divided into three subtypes with different prognoses, and an OSPM-related signature including eight OSPM-related genes (i.e., SLC2A1, MMP14, TOP2A, MBOAT2, ANLN, ECT2, SLC22A3, and FGD6) was developed. High- and low-risk subgroups divided by the signature showed different prognoses, expression levels of immune checkpoint genes, immune cell infiltration, and tumor microenvironment. The risk score was negatively correlated with the proportion of TIL, pDC, Mast cell, and T cell co-stimulation. The expression levels of genes in the signature were verified in PC and normal samples. The protein levels of SLC2A1, MMP14, TOP2A, MBOAT2, ANLN, and SLC22A3 showed up-regulation in PC samples compared with normal tissues. After integrating metabolomics and transcriptomics data, the alterations in OSPM in PC were investigated, and an OSPM-related signature was developed, which was helpful for the prognostic assessment and individualized treatment for PC.

## Introduction

Pancreatic cancer (PC), a gastrointestinal malignancy, represents nearly 95% of pancreatic cancer cases^1,2^. It is widely recognized as a lethal malignancy. The 5-year survival rate of PC is about 10%^3^. Surgery is currently the main treatment for PC, but it is not suitable for patients with advanced disease. Although some patients with PC can benefit from radiotherapy and chemotherapy, the overall survival is still poor^4^. To date, few genetic mutations have been demonstrated to be connected with the occurrence of PC, and no clear mechanisms for the development of PC have been investigated. For a reduction in PC mortality, early diagnostic biomarkers and effective therapeutic targets are urgently needed^5^.

Oxidative stress (OS), with the overproduction of reactive oxygen species (ROS), is reported to be associated with oncogenesis^6,7^. ROS is produced in the process of mitochondrial respiration and is reported to be essential in signaling pathways^8^. The level of oxidative stress is linked to the oxidizing capacity and the antioxidation power. Excess ROS damages to cell components, including lipids^9^. Targeting OS is considered a novel treatment modality in various diseases, including cancer^10^. Current evidences support that the dysfunction of ROS is a risk factor and antioxidant supplementation is a preventive method for PC^11,12^.

Lipids, including triglycerides, cholesterol, and phospholipids, are essential for cancer metabolism and the activation of immune cells^13,14^. Currently, it is reported that lipid metabolic reprogramming including the increase of lipid uptake, storage and lipogenesis can result in rapid tumor growth and is viewed as a newly recognized hallmark of malignancy^15^. Lipid metabolic reprogramming can influence the biological activities of cancer cells and is reported to be of vital importance in solid tumors^13,16^. Phospholipids, the major component of cell membranes, are significant for cell structure and metabolism^17^. Additionally, phospholipids have been proposed as the inducers of cancer multidrug resistance^18^. Overall, oxidative stress, which compromises lipid function impairs and is related to lipid metabolic reprogramming, was found to function in the development and metastasis of cancer^7,19,20^. The disorders of phospholipid affect the development and drug sensitivity of PC^21,22^. The specific roles of OS and phospholipid metabolism (PM) in PC has not been investigated clearly.

By integrating genomic, transcriptomic, proteomic, and metabolomic data, multi-omics studies have revealed the discovery of novel tumor prognostic markers^23^. Previous findings offer fresh insights into clinical prognostic assessment by providing valuable bioinformatics model^24,25^. Moreover, the utilization of machine learning algorithms on multi-omics data has demonstrated promising predictive capabilities in identifying tumor prognostic markers, which holds significant potential in delivering more accurate guidance for personalized treatment and survival prediction^26,27^. In this study, the altered metabolites in the occurrence of PC were comprehensively explored and the potential impacts of OSPM in the prognosis of PC were investigated. The significant genes in this process of OSPM were summed up from a pan-cancer perspective. Based on the function of OSPM, PC samples were classified into OSPM-active, OSPM-normal, and OSPM-inactive subtypes. Samples in the OSPM-active subtype had a poor prognosis. The specific alterations and discrepancies in the OSPM-active subtype were explored. Furthermore, an OSPM-related signature was constructed to identify the risk of PC patients. After detailed exploration and demonstration, the OSPM-related signature showed promising prognostic performance and diagnostic accuracy.

## Methods

### Data acquisition

The metabolic data of 35 pancreatic tumor samples, 34 para-carcinoma samples, and 31 normal pancreatic tissues were obtained from the previously published literature, completed by Liu et al.^28^. In total, 10,028 OS-related genes and 4,479 PM-related genes were searched in the Molecular Signatures Database (MSigDB)^29^ and GeneCard websites^30^. Specifically, the GeneCard platform provided 10,022 OS-related genes and 4477 PM-related genes. MSigDB platform provided two OS-related pathways (i.e. *GOBP_RESPONSE_TO_OXIDATIVE_STRESS*, *WP_OXIDATIVE_STRESS_RESPONSE*) and two PM-related pathways (i.e. *GOBP_REGULATION_OF_PHOSPHOLIPID_METABOLIC_PROCESS*, *REACTOME_PHOSPHOLIPID_METABOLISM*). “GOBP_RESPONSE_TO_OXIDATIVE_STRESS” pathway involves 437 OS-related genes, and “WP_OXIDATIVE_STRESS_RESPONSE” pathway involves 33 OS-related genes. “GOBP_REGULATION_OF_PHOSPHOLIPID_METABOLIC_PROCESS” pathway involves 37 PM-related genes, and “REACTOME_PHOSPHOLIPID_METABOLISM” pathway involves 211 PM-related genes. After removing duplicate genes, a final set of 10,028 OS-related genes and 4479 PM-related genes was obtained.

The gene expression data and clinical information were obtained from public databases. In total, PC datasets contained 930 PC samples made up of GSE28735, GSE57495, and GSE62452 from the Gene Expression Omnibus (GEO), MTAB-6134 from ArrayExpress, TCGA-PC samples from The Cancer Genome Atlas (TCGA), and ICGC-AU and ICGC-CA datasets from International Cancer Genome Consortium (ICGC). The Genotype-Tissue Expression (GTEx) database was searched to obtain normal pancreatic samples.

For pan-cancer summarization, mRNA expression, clinical information, single-nucleotide variation (SNV), copy number variation (CNV), and methylation data of common cancers were downloaded from TCGA database.

### Identification of metabolic reprogramming in the occurrence of PC

Liu et al. detected and analyzed the metabolites in PC and presented the metabolic reprogramming in PC. We downloaded the published literature compiled by Liu et al., which contained the metabolomics data of normal and tumor tissues as supplementary materials^28^. This study involves the secondary mining of metabolomics data based on the supplementary materials provided in the article by Liu et al. After collecting and analyzing the metabolomics data, the alterations in metabolites caused by the occurrence of PC were further investigated. The detailed approaches are described as follows. The metabolic changes were investigated, and each fold change (FC) value was calculated after comparing tumor tissues with para-carcinoma tissues. Meanwhile, the differences between tumor tissues and normal tissues were also explored. On the basis of the p.adjust < 0.05 and FC > 2 or FC < 0.5, the metabolites with different contents in PC were obtained.

### Identification of key OSPM-related genes in PC

Considering the prominent roles of OS and PM in the development of PC, we integrated the gene sets corresponding to OS and PM, resulting in the formation of OSPM genes. Then the OSPM-related differentially expressed genes (DEGs) were explored in three cohorts, namely GSE28735 (cohort1), GSE62452 (cohort2), and TCGA dataset combined with the GTEx dataset (cohort3). All the DEGs (criteria: FC > 2 or FC < 0.5) acquired from the three cohorts were taken into intersection. For further investigation of the prognostic values of the OSPM-related DEGs, univariate Cox regression analysis and Kaplan–Meier (KM) survival analysis were conducted. The prognostic OSPM-related DEGs were collected as candidate genes for the subsequent analyses.

### The role of OSPM in pan-cancer

Given the potential function of OSPM in various cancers and the essential role of the OSPM-related genes in the process of OSPM, a comprehensive investigation of the OSPM-related genes was made in pan-cancer. First, the alteration in the expression level was assessed according to the FC, and the prognostic impact was distinguished by conducting univariate Cox regression analysis. Next, the CNV (amplified or deleted) was summed up. As for SNV, the mutation frequency (samples with SNV/all samples) was calculated and the detailed mutation types were exhibited. Then the status of promoter methylation in tumor samples was obtained after comparing with normal samples in pan-cancer. All the methods have been utilized and described before^31–33^.

### OSPM-based cluster analysis

With the mRNA expression of the OSPM-related genes in PC, the function states of OSPM were estimated quantitatively with ssGSEA algorithm. Whether the discrepancies of OSPM could influence the survival of the sample was investigated after performing cluster analysis. During the process, “gplots” and “pheatmap” packages in R were employed to visualize the results of cluster analysis and relative expression levels of each OSPM-related genes^34^. Based on the ssGSEA scores obtained in the normal samples, PC samples were grouped into cluster1 with OSPM-active function, cluster2 with OSPM-inactive function, and cluster3 with OSPM-normal function.

Then the discrepancies in the different clusters were searched by performing the following analyses^35,36^: (1) Comparing the OSPM function scores by the Kruskal–Wallis test; (2) Comparing the survival probability by KM analysis; (3) Comparing expression levels of tumor suppressor and promoter genes through the Kruskal–Wallis test; and (4) Comparing the expression levels of immunological checkpoint genes (ICGs) by the Kruskal–Wallis test.

The estimation of the tumor immune microenvironment was conducted as follows ^37^: (1) ssGSEA was performed to quantify the immune-associated gene sets; (2) Spearman's correlation analysis was conducted determine the link between prognostic OSPM-related DEGs and immune cells; (3) The correlation between the OSPM function score and immune components was found by utilizing “ggstatsplot,” “data.table,” “dplyr,” “tidyr,” and “ggplot2” packages in R; and (4) MCPCOUNTER, XCELL, CIBERSORT, EPIC, CIBERSORT-ABS, and TIMER were performed to assess immune responses, and the statistically significant discrepancies among different clusters were exhibited.

### Group division and OSPM-related signature construction

To develop a quantitative signature for guiding the clinical diagnosis and treatment of PC, an OSPM-related signature was constructed and validated. The training cohort (635 PC samples) included GSE57495, GSE28735, GSE62452, MTAB-6134, and TCGA-PC datasets, whereas the validation cohort (295 PC samples) included the ICGC-CA and ICGC-AU datasets. To make the signature concise and convenient, random survival forest analysis was conducted using the “randomForestSRC” package in R to select the most appropriate genes from all the prognostic OSPM-related DEGs to gain an optimal prognostic signature^38^. Multivariate Cox regression analysis was then conducted, and the coefficients helped to obtain the risk score of each sample^39^.

The risk score corresponding to each sample was determined, and the median score helped to distinguish high- and low-risk subgroups in the training and test cohorts, respectively. Then the discrepancies of the high- and low-risk subgroups were investigated in the two cohort as follows: (1) Sample division was visualized through principal component analysis (PCA) and t-distributed stochastic neighbor embedding (t-SNE); (2) Survival probability was compared through KM analysis^40,41^; (3) The expression levels of the genes in the signature were compared between high- and low-risk subgroups and exhibited in heatmap; (4) The diagnostic accuracy was demonstrated by developing time-dependent receiver operating characteristic (ROC) curves and obtaining the AUC values^42^; (5) The expression levels of ICGs were compared, and the immune response was estimated through the methods mentioned above^43,44^. In addition, the GEPIA website was utilized to compare the expression levels of the model genes in PC samples with different clinical stages^45^.

### Cell culture and qRT-PCR

Human pancreatic cell line (HPDE6-C7) and PC cell lines (BxPC-3, CFPAC-1, Panc-1, and Mia-Paca-2) were obtained for PCR experiments of model genes. HPDE6-C7, Panc-1, and Mia-Paca-2 were cultured in DMEM medium. BxPC-3 was maintained in 1640 medium. CFPAC-1 was maintained in IMDM medium. All these mediums were supplemented with 10% fetal bovine serum at 37 °C and 5% –CO_2_. Total RNA was extracted utilizing TRIzol reagent and synthesized into cDNA utilizing M-MLV reverse transcriptase under the instruction of the manufacturer. qRT-PCR was performed using SYBR Green assay and β-actin (Forward: CCTGGGCATGGAGTCCTGTG, Reverse: TCTTCATTGTGCTGGGTGCC) was selected as an endogenous reference. The specific PCR primer sequences are as follows: SLC2A1 (FORWARD: ATGAAGGAAGAGAGTCGGCAGATG, REVERSE: AGCACCACAGCGATGAGGATG), MMP14 (FORWARD: TGCGTCCATCAACACTGCCTAC, REVERSE: CGCCTCATCAAACACCCAATGC), TOP2A (FORWARD: GCACCAGCACATCAAAGGAAGC, REVERSE: ATAGCAGCATCATCTTCAGGACCAG), MBOAT2 (FORWARD: CCATCTCCAAATACTGCGGTTGTTC, REVERSE: GCTCATCAATGTTGTACTCCACAGG), ANLN (FORWARD: GTTCTCCAAGTCCTGTGTCTCCTC, REVERSE: TGCAGTTGCTTCCAATCTTGAGTTC), ECT2 (FORWARD: AGCTCCACTCCAGTTCCTTCAAAG, REVERSE: GGTCCACCACGTTGTCCTTCC), SLC22A3 (FORWARD: CGTGTGGCTAGAACTACCTCTGATC, REVERSE: ACTGTCTCTGGCAAGGCAATACC), FGD6 (FORWARD: CAACTCGGAGACACCACCACAG, REVERSE: CTACCAACAAGCCTTGCCAATCAC).

### Single-cell expression level, immunohistochemistry, and immunofluorescence of model genes in PC

Tumor samples are composed of a variety of cells. Tumor Immune Single-cell Hub 2 (TISCH2), a scRNA-seq database focusing on tumor microenvironment (TME), was searched for identifying of major cell types and the expression levels of model genes in different cells in PC^46^. The Human Protein Atlas (HPA: https://www.proteinatlas.org/) was utilized for further exploration about the protein levels of model genes^47,48^. Additionally, the HPA was also utilized to exhibit the cellular localization on the basis of immunofluorescence.

## Results

### Identification of metabolic alterations and OSPM-related genes in PC

**Figure 1:**
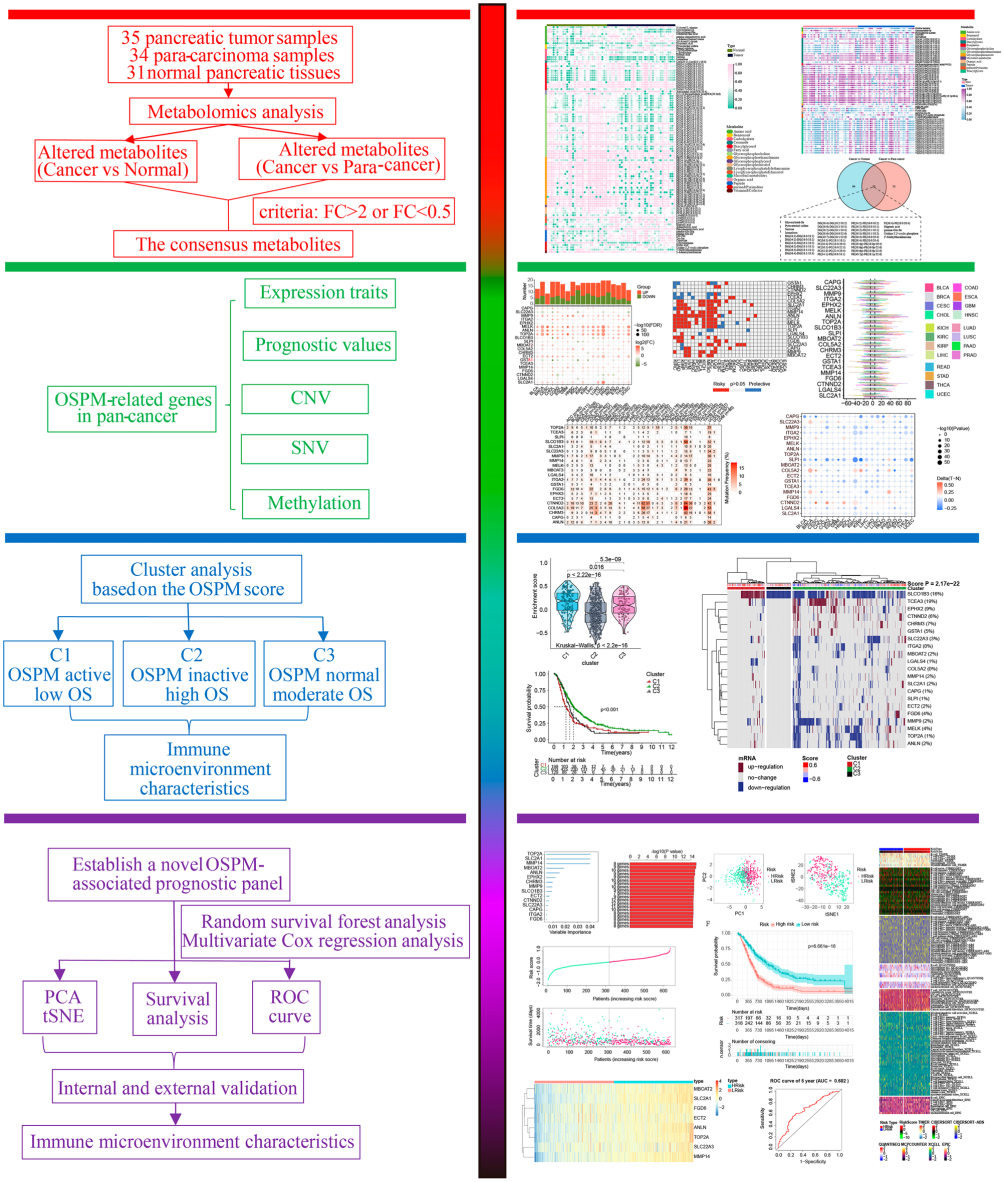
Flow chart of the study.

**Figure 2:**
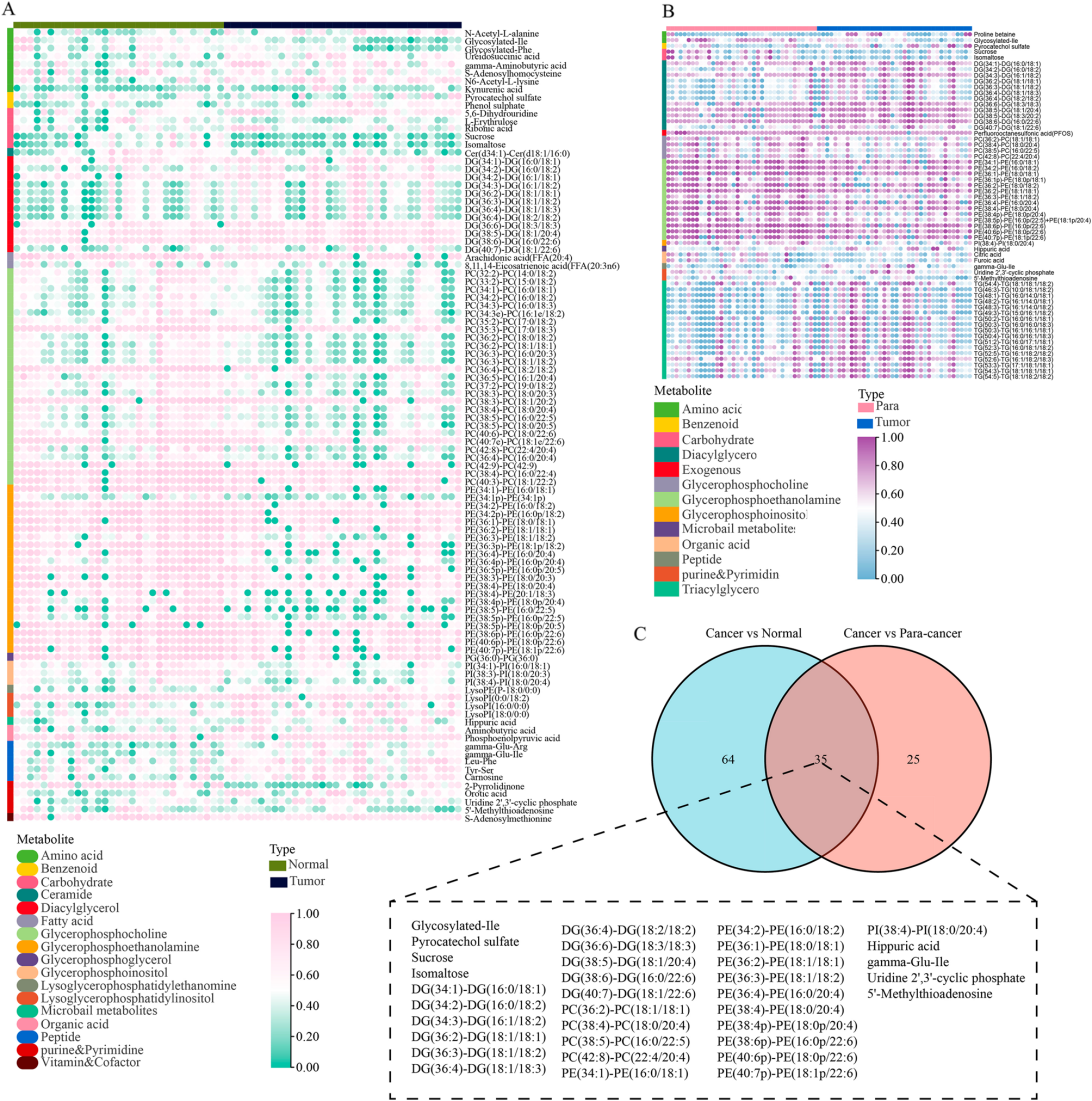
Metabolomics analyses for exploring the altered metabolites. (**A**) The altered metabolites (Cancer vs. Normal); (**B**) The altered metabolites (Cancer vs. Para-cancer); (**C**) The common differential metabolites. The heatmaps were created with the help of TBtools (version 1.120, https://bio.tools/tbtools).

The workflow is depicted in Fig. 1. The alteration of metabolites plays a vital role in tumor initiation and progression. The discrepancies of the content of metabolite between pancreatic tumor samples and para-carcinoma samples were investigated, and 99 metabolites with significant changes were exhibited in the heatmap (Fig. 2A). Similarly, 60 metabolites altered significantly between tumor samples and normal samples, as shown in another heatmap (Fig. 2B). After taking an intersection, 35 shared metabolites were obtained (Fig. 2C). Of note, the shared metabolites primarily belonged to diacylglycerol, glycerophosphocholine, and glycerophosphoethanolamine. It is well-known that PM mainly includes the metabolism of glycerophospholipids and sphingophospholipids. And diacylglycerol is the major substrates for the synthesis of phosphatidylglycerol in glycerophospholipid metabolism. Conversely, glycerophospholipids can be converted into glycerophosphocholine and glycerophosphoethanolamine by the action of phospholipase B, after removing the fatty acids. As a result, PM showed a proper role in the development of PC. Due to the influence of OS on PM, the following investigations were mainly focused on the two biological processes.

**Figure 3:**
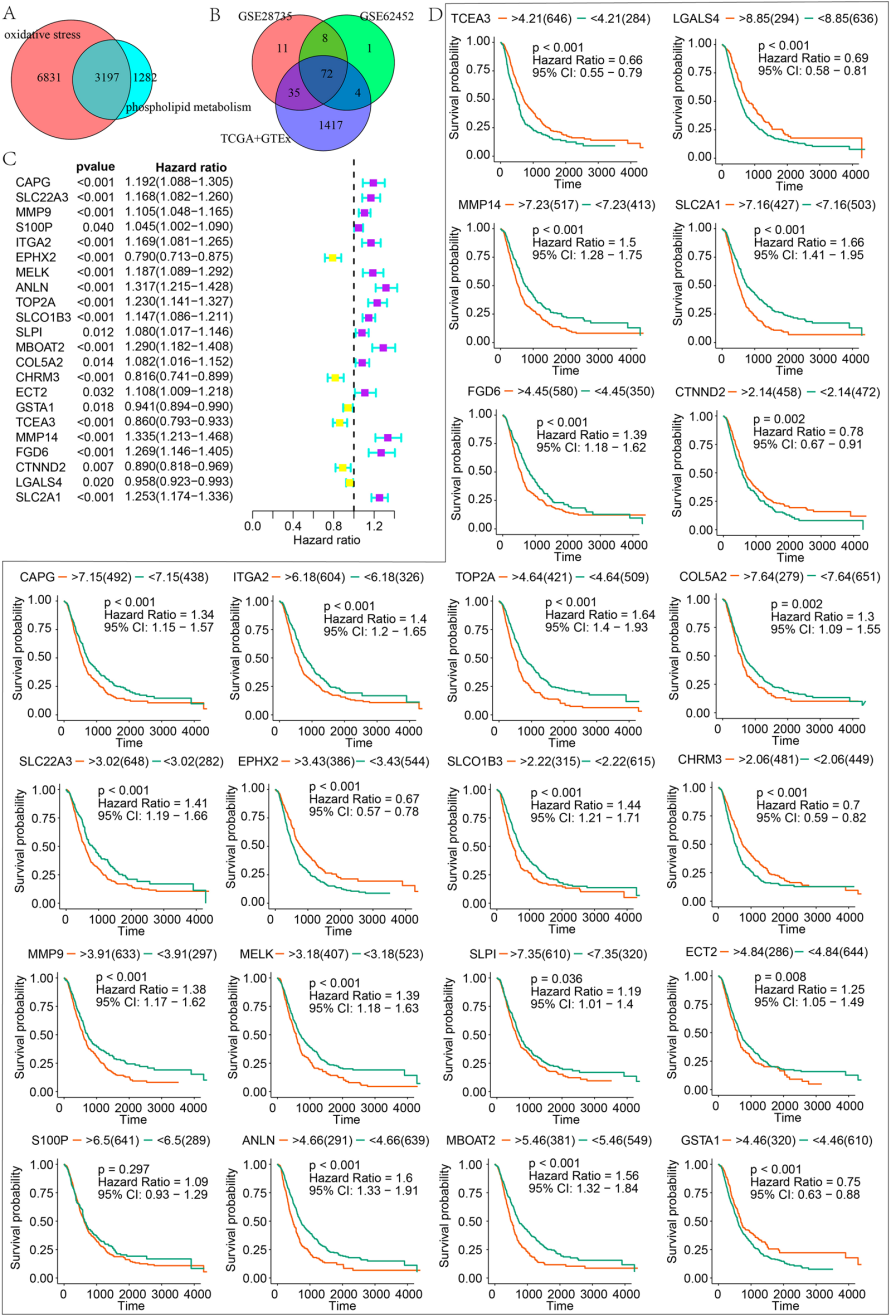
Identification of key OSPM-related genes in PC. (**A**) The intersection of OS-related genes and PM-related genes; (**B**) The common OSPM-related differentially expressed genes (DEGs); (**C**,**D**) Univariate Cox regression analysis and Kaplan–Meier survival analysis for demonstrating the prognostic value of OSPM-related DEGs.

After taking an intersection of the 10,028 OS-related genes and 4,479 PM-related, 3,197 OSPM-related genes were used in the following analyses (Fig. 3A). Then DEGs between tumor samples and normal samples were explored in GSE28735 (cohort1), GSE62452 (cohort2), and TCGA dataset combined with GTEx dataset (cohort3; Supplementary Figs. 1–3). In total, 72 intersected OSPM-related DEGs were obtained (Supplementary Table 1; Fig. 3B). Univariate Cox regression analysis was conducted, and 22 prognostic OSPM-related DEGs were selected (Fig. 3C). KM survival analysis was performed for further demonstration of the prognostic value of these DEGs. In total, 21 prognostic OSPM-related DEGs were screened as candidate genes (Fig. 3D).

### The role of OSPM in pan-cancer

**Figure 4:**
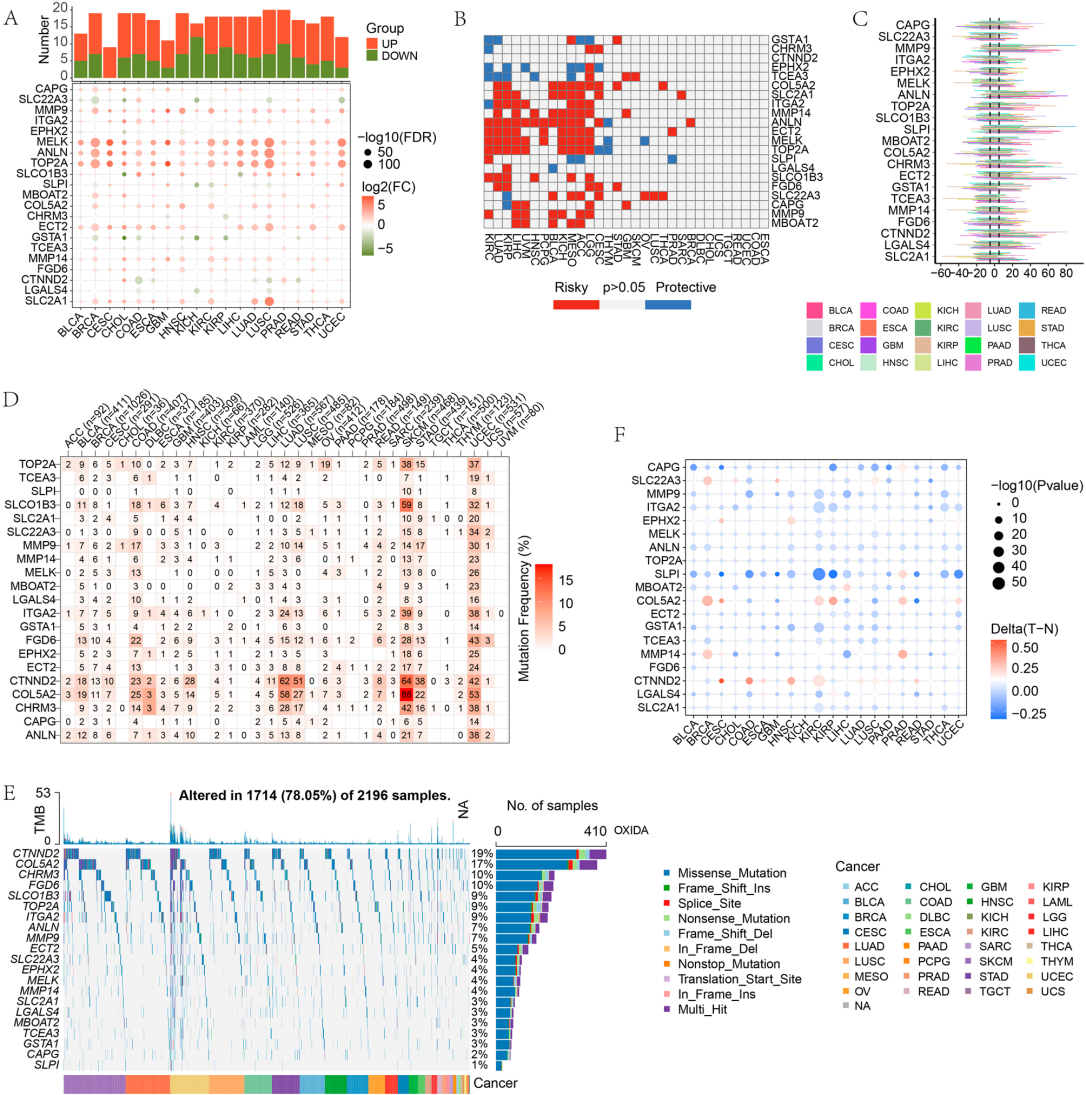
OSPM-related genes in pan-cancer. The mRNA alteration (**A**), the prognostic value (**B**), the CNV frequency (**C**), the SNV frequency (**D**), the mutation types (**E**), and status of promoter methylation (**F**) of OSPM-related genes. The heatmaps were created with the help of R language (version 4.0.3, https://www.r-project.org/).

Due to the significant role of the 21 prognostic OSPM-related DEGs, they were viewed as the functional genes in the OSPM progress. From a pan-cancer perspective, the general summarization of the 21 OSPM function genes was made. First, the mRNA levels of these genes in tumor samples were compared with those in normal samples. MELK, ANLN, and TOP2A were markedly up-regulated in almost all types of tumors (Fig. 4A). Then the prognostic performances of the 21 genes were explored and exhibited in the heatmap. Genes with hazard ratio (HR) > 1 and p < 0.05 were risky genes, while those with HR < 1 and p < 0.05 were protective genes (Fig. 4B). The CNV and SNV characteristics of 21 genes in pan-cancer were displayed in Fig. 4C–E. Among all types of tumor samples, LUAD, LUSC, SKCM, STAD, and UCEC patients exhibited the most prominent SNV phenomena (Fig. 4D). Additionally, among the 21 genes, CTNND2 showed the highest mutation rate in these cancers, and missense mutation was the most common mutation type (Fig. 4E). Promoter methylation levels revealed that CTNND2 exhibited hypermethylation in CESE, COAD, and HNSC. Conversely, SLPI showed hypomethylation in nearly all cancers, with the exception of PRAD (Fig. 4F).

### OSPM-based cluster analysis

**Figure 5:**
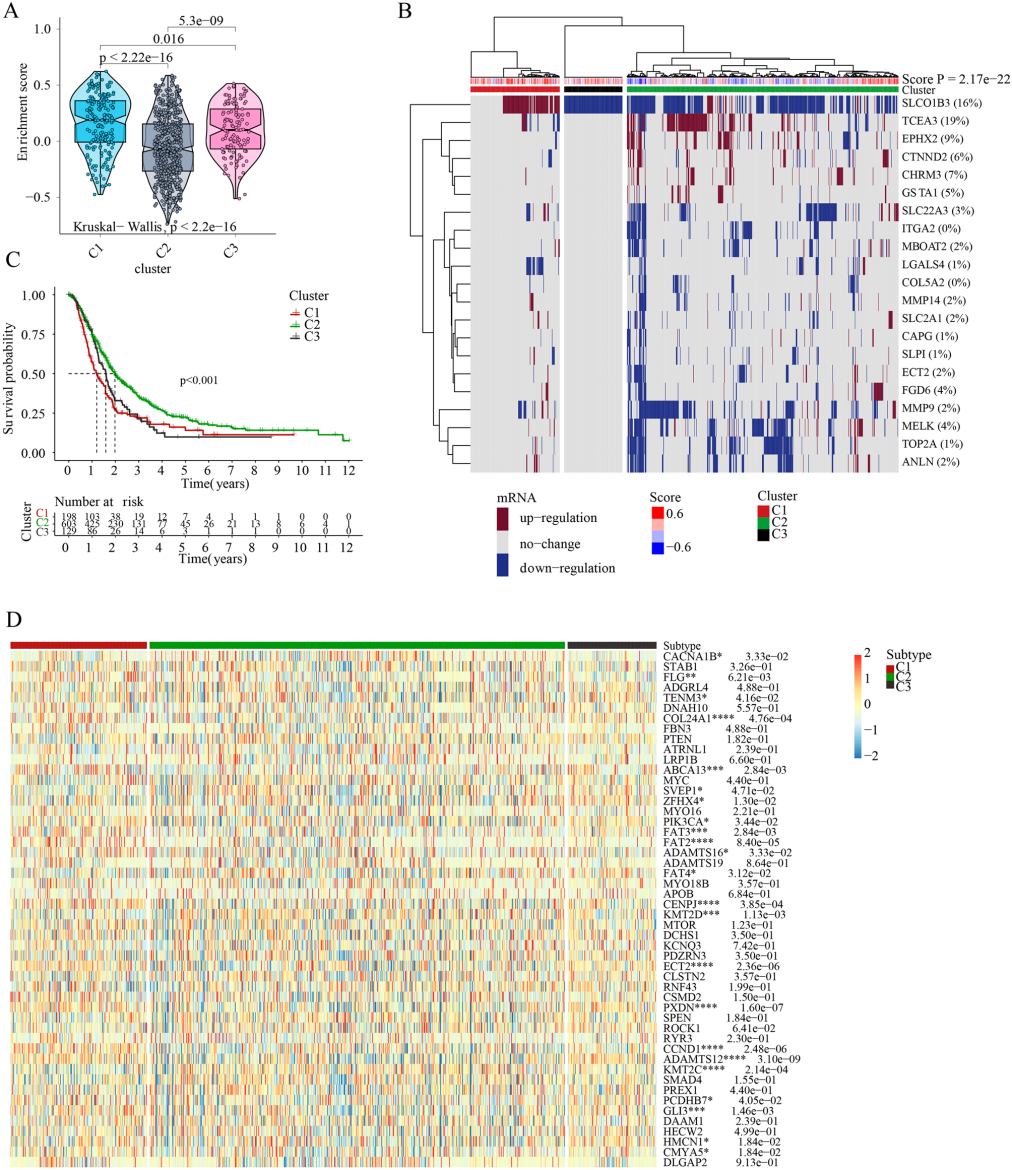
OSPM-based cluster analysis. The discrepancies of OSPM function scores (**A**), OSPM function genes’ expressions (**B**), survival probabilities (**C**), and the expression of tumor suppressor genes and oncogenes in three clusters (**D**). The heatmaps were created with the help of R language (version 4.0.3, https://www.r-project.org/).

**Figure 6:**
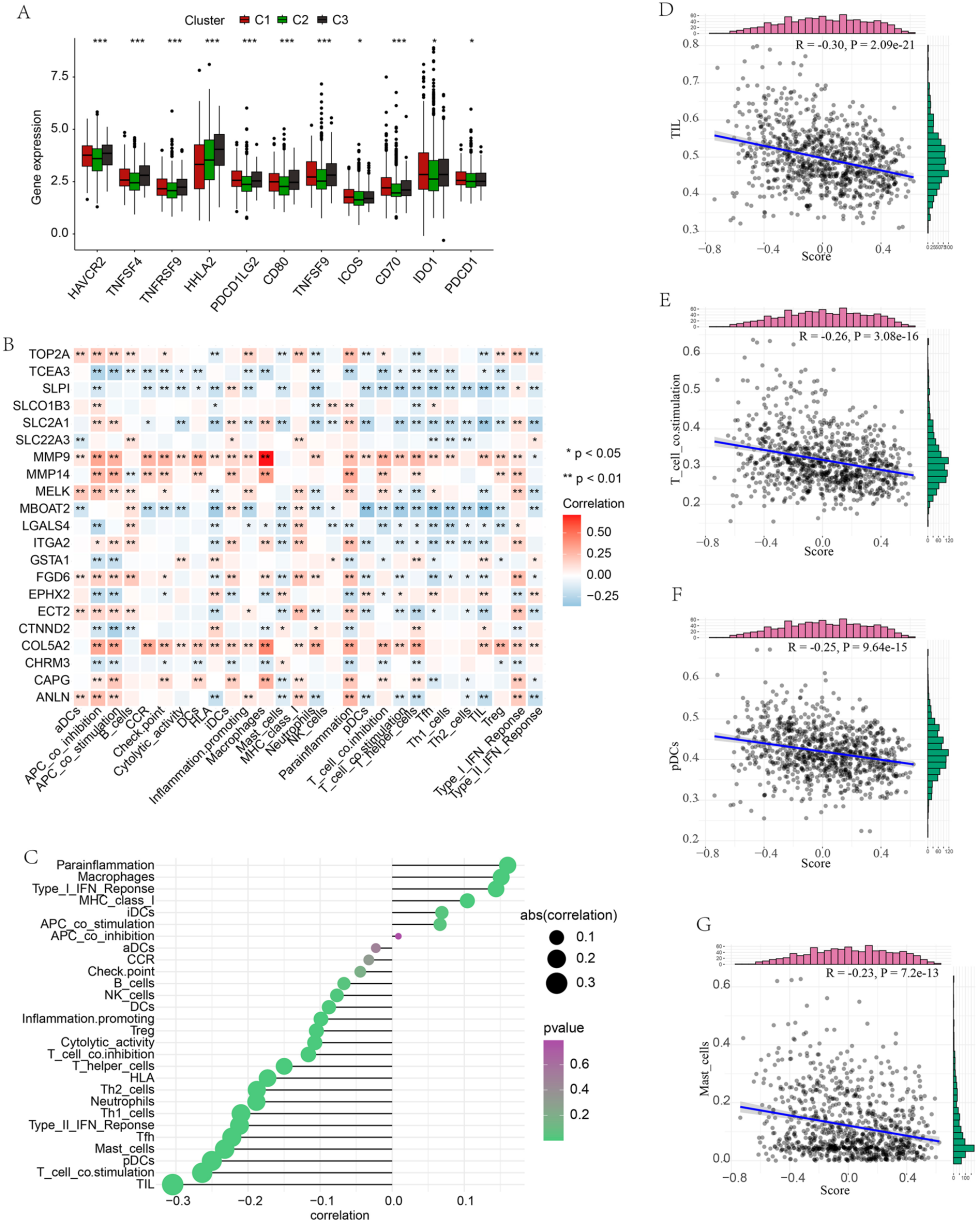
The OSPM-related alteration of immune status in PC. (**A**) Comparing the expression of ICGs in the three PC subtypes; (**B**) The link between OSPM-related genes and immune cells; (**C**) The correlation between the OSPM function score and immune components; The correlation between OSPM function score and the proportion of TIL (**D**), T cell co-stimulation (**E**), pDC (**F**), and Mast cell (**G**).

To further illustrate the role of OSPM in PC, the ssGSEA was performed using a score derived from the expression of 21 OSPM function genes. Based on the enrichment scores in normal samples, 930 PC samples were divided into three clusters (C1, C2, and C3). Subsequently, the enrichment scores of the OSPM function were compared. C1, C2, and C3 were the OSPM-active, OSPM-inactive, and OSPM-normal cluster (scores: C1 > C3 > C2; Fig. 5A). The heatmap presented herein showcases the expression levels of the 21 OSPM function genes (Fig. 5B). For example, SLCO1B3 exhibited a clear upregulation in C1 and downregulation in C2 and C3 (Fig. 5B). The survival probabilities in the three clusters demonstrated statistically significant differences, with C2 showing the highest survival probability, followed by C3, and then C1 (Fig. 5C). Regarding the expression of tumor suppressor genes and oncogenes, COL24A1, FAT2, CENPJ, ECT2, PXDN, CCND1, and ADAMTS12 exhibited elevated expression in C1. Conversely, KMT2C displayed increased expression specifically in C2 (Fig. 5D).The differential expressions of ICGs might also contribute to the prognosis of pancreatic cancer (PC). HAVCR2, TNFSF4, TNFRSF9, PDCD1LG2, CD80, TNFSF9, ICOS, CD70, IDO1, and PDCD1 exhibited significantly higher expression in C1 compared to C2. Conversely, HHLA2 displayed reduced expression in C1 (Fig. 6A). Furthermore, the immune response showed distinct variations among the three clusters. Following the application of various algorithms, the infiltration patterns of immune cells, which exhibited statistically significant differences, are illustrated in the heatmap presented herein(Supplementary Fig. 4). The relationship between the expression of the 21 OSPM function genes and the infiltration of immune cells was illustrated in Fig. 6B. The findings revealed that MMP9, MMP14, and COL5A2 exhibited positive correlations with nearly all types of immune cells, while TCEA3, SLPI, and MBOAT2 displayed negative correlations with these immune cells. To provide a concise overview of the immune response, Spearman's correlation analysis was conducted. The results indicated a negative correlation between the OSPM function score and the proportion of tumor-infiltrating lymphocytes (TILs) (r = − 0.30, p = 2.09e−21), T cell co-stimulation (r = − 0.26, p = 3.08e−16), pDC (r = − 0.25, p = 9.64e−15), and Mast cell (r = − 0.23, p = 7.20e−13; Fig. 6C–G).

### Identification of the OSPM-related signature

**Figure 7:**
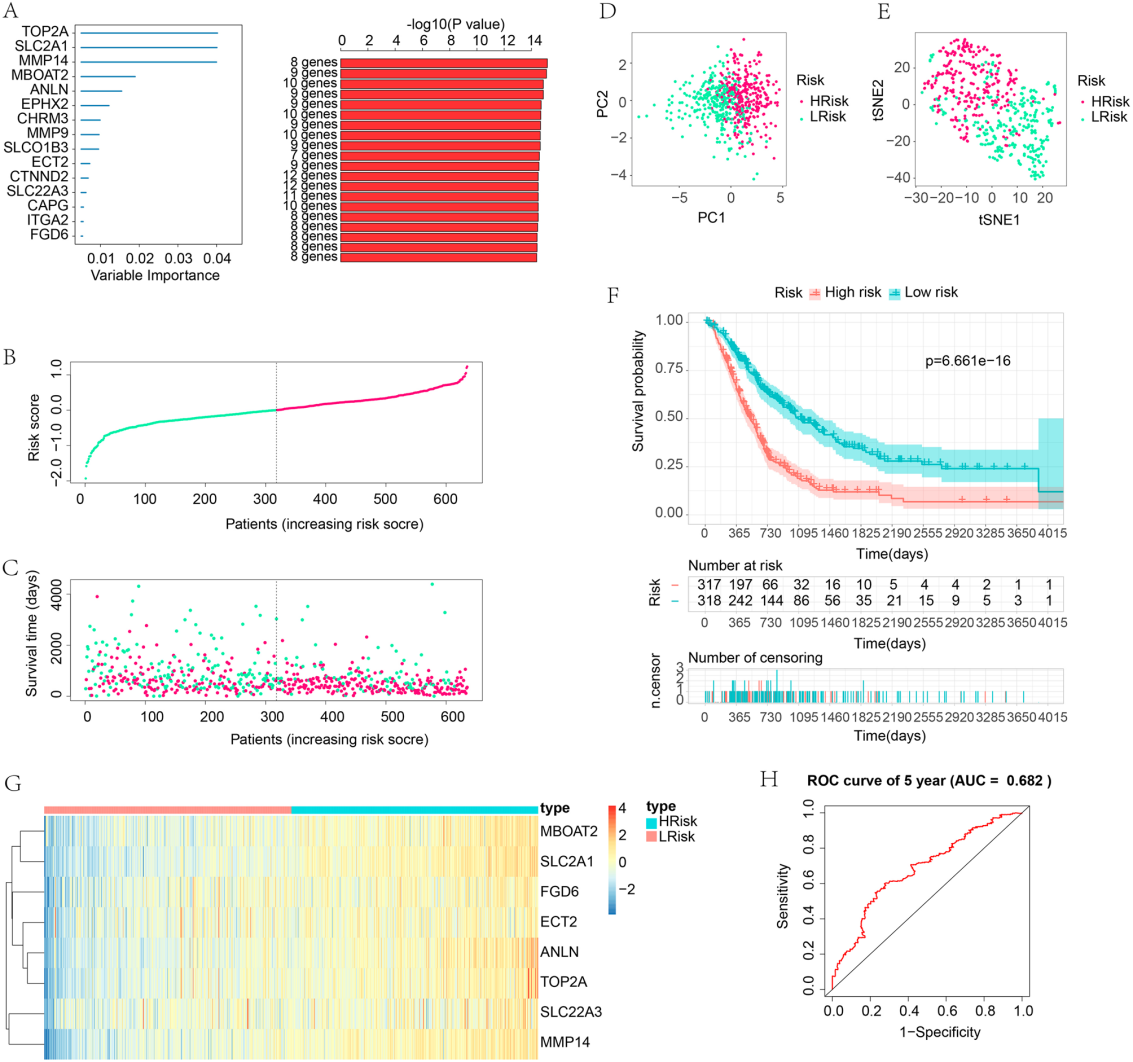
Identification of the OSPM-related signature in the training cohort. (**A**) Random survival forest analysis for the selection of the most appropriate genes combination; (**B**) Group division of PC samples; (**C**) The link between risk score and survival; (**D**) PCA analysis; (**E**) t-SNE analysis; (**F**) The survival analysis in the high- and low-risk subpopulations; (**G**) Heatmap exhibiting the expression of the signature genes; (**H**) ROC curves for demonstrating the diagnostic accuracy of the signature. The heatmaps were created with the help of R language (version 4.0.3, https://www.r-project.org/).

For correct establishment and complete validation of the OSPM-related signature, the training and test cohorts were grouped. In the training cohort, random survival forest analysis helped identify the most appropriate genes (i.e., SLC2A1, MMP14, TOP2A, MBOAT2, ANLN, ECT2, SLC22A3, and FGD6) related to survival of PC patients, and an OSPM-related signature was identified (Fig. 7A). The following multivariate Cox analysis helped identify the risk score of each sample. The computational formula of risk score is as follows. The risk score = 0.229994697234363*TOP2A + 0.124787795719623*SLC2A1 + 0.220093179394101*MMP14 + 0.0671974510904209*MBOAT2 + 0.0484008588233876*ANLN − 0.17194413420108*ECT2 + 0.0362333570976207*SLC22A3 + 0.0192255505103104*FGD6. The median risk score of training cohort is 0.002583997. Based on the median risk score, samples were grouped into high- and low-risk subgroups (Fig. 7B and C). The PCA and t-SNE demonstrated the distinction between the two subgroups (Fig. 7D and E). The survival analysis indicated that samples with high-risk scores had poor prognoses (Fig. 7F). The eight genes in the OSPM-related signature showed different expression in the two subgroups. All these genes had increased expression in the high-risk samples (Fig. 7G). Interestingly, the expression of ECT2, FGD6, MMP14, and SLC2A1 were statistically different in the patients with different tumor stage, which implied that ECT2, FGD6, MMP14, and SLC2A1 might be associated with the development of PC (Supplementary Fig. 5). The AUC value of the ROC curve was 0.682 for 5-year survival in the training cohort, which implied its accurate diagnosis (Fig. 7H). The immune responses in high- and low-risk subgroups also exhibited discrepancies. As is depicted in the heatmap, the infiltration of various immune cells in the two subgroups differed from each other, which is one of the root causes of the prognosis (Supplementary Fig. 6A). Furthermore, the expression level of the ICG might be another root cause of the prognosis of PC. As depicted in Supplementary Fig. 6B, SIGLEC15, HAVCR2, PDCD1LG2, YTHDF1, CD274, CD80, TNFRSF14, TNFSF4, TNFRSF9, and CD70 showed increased expression in the high-risk subgroup.

**Figure 8:**
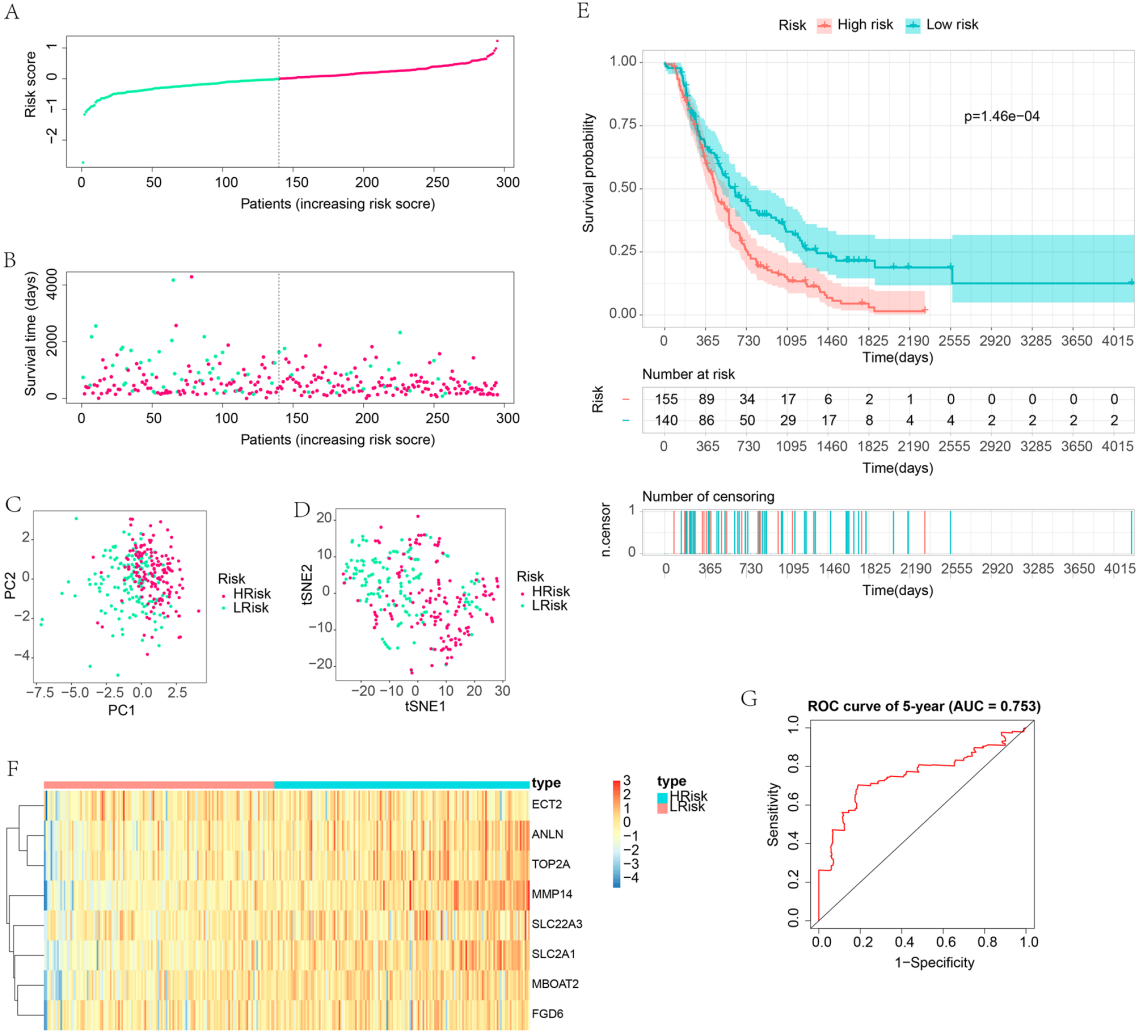
Validation of the OSPM-related signature in the test cohort. (**A**) Group division; (**B**) The link between risk score and survival; (**C**) PCA analysis; (**D**) t-SNE analysis; (**E**) The survival analysis in the high- and low-risk subpopulations; (**F**) Heatmap exhibiting the expression of the signature genes; (**G**) ROC curves for demonstrating the diagnostic accuracy of the signature. The heatmaps were created with the help of R language (version 4.0.3, https://www.r-project.org/).

To validate the performance of the OSPM-related signature, samples in the test cohort were grouped into high- and low-risk subgroups according to the median risk score of 0.002583997 (Fig. 8A and B). The analyses described above were performed again in the test cohort. Samples in high- and low-risk subgroups also showed distinct separation through PCA and t-SNE (Fig. 8C and D). High-risk subpopulations also had a poor prognosis (Fig. 8E). All the genes in the signature showed increased expression in the high-risk subgroup (Fig. 8F). The AUC value of the ROC curve in the test cohort were 0.753 for 5-year survival (Fig. 8G). Also, the immune responses in the test cohort are depicted in Supplementary Fig. 6C. The expression of ICGs showed a similar trend in the test cohort. TIGIT, HAVCR2, PDCD1LG2, TNFRSF4, TNFSF4, TNFRSF9, and CD70 were up-regulated in the high-risk subgroup in the test cohort (Supplementary Fig. 6D). All these results in the test cohort were similar to those in the training cohort, which indicated the satisfactory performance of the OSPM-related signature.

### The expression of the eight genes in the OSPM-related signature

**Figure 9:**
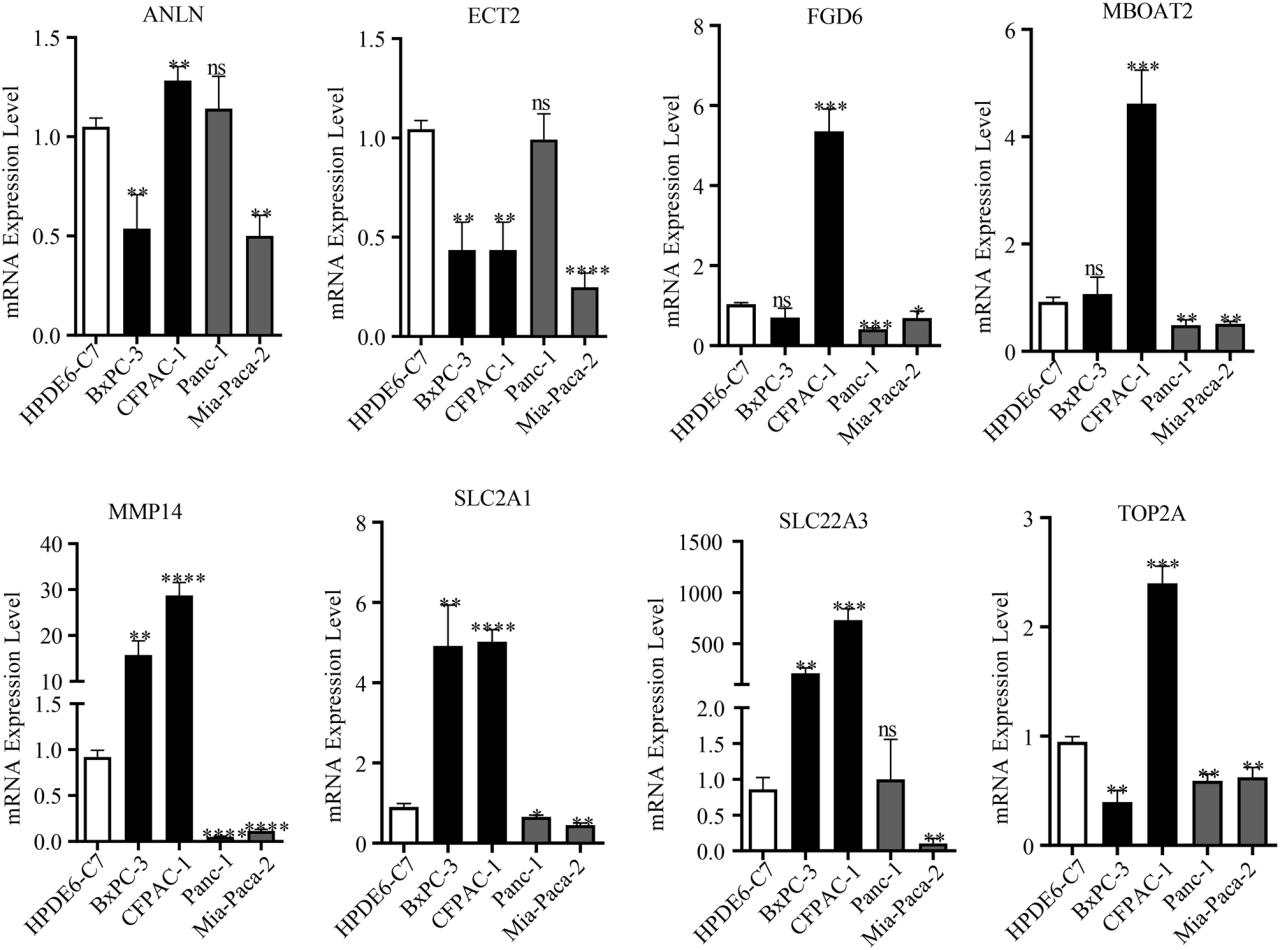
qRT-PCR for the investigation about the expression of the eight genes in the OSPM-related signature.

For further investigation of the eight genes (i.e., SLC2A1, MMP14, TOP2A, MBOAT2, ANLN, ECT2, SLC22A3, and FGD6) in the OSPM-related signature, the qRT-PCR was conducted. And the results indicated that the expression levels of these genes in PC were different with that in normal samples. For ANLN, its expression was higher in CFPAC-1 while lower in BxPC-3 and Mia-Paca-2. For ECT2, its expression was lower in CFPAC-1, BxPC-3 and Mia-Paca-2. For FDG6 and MBOAT2, the expression levels were higher in CFPAC-1 while lower in Panc-1 and Mia-Paca-2. For MMP14 and SLC2A1, the expression levels were higher in BxPC-3 and CFPAC-1 while lower in Panc-1 and Mia-Paca-2. For SLC22A3, its expression was higher in BxPC-3 and CFPAC-1 while lower in Mia-Paca-2. For TOP2A, its expression was higher in CFPAC-1 while lower in BxPC-3, Panc-1 and Mia-Paca-2 (Fig. 9).

### Single-cell expression level, immunohistochemistry, and immunofluorescence of model genes in PC

To investigate the tumor microenvironment, single-cell expression levels of the eight genes in the signature were explored. The expression of MBOAT2 primarily existed in the epithelium and the expression of MMP14 mainly existed in fibroblasts, while the expression of FGD6 primarily existed in the progenitor (Supplementary Fig. 7A). Regarding the protein levels of these genes, SLC2A1, MMP14, TOP2A, ANLN, and SLC22A3 exhibited higher expression in tumor samples compared to normal samples. Additionally, immunofluorescence helped determine the cell location of these proteins (Supplementary Fig. 7B).

## Discussion

PC, as a pancreatic malignancy, is projected to be the second leading cause of cancer-related deaths by 2030^49^. Owing to the lack of a reliable diagnostic method, most PC patients are diagnosed at an advanced stage^50^. The incidence of pancreatic cancer was reported to be around 57,600 with 47,050 deaths in the United States in 2020^51^. Surgery is now the only curative treatment method for PC; therefore, the underlying cause and precise mechanisms of PC needed to be explored. Searching for more effective biomarkers for the diagnosis and treatment of PC patients is urgent. OS is the imbalance of the production and clearance of ROS. High levels of ROS lead to severe oxidative damage in proteins, lipids, and DNA^52–54^. Phospholipids, are especially susceptible to ROS attack^55,56^. It has been discovered that OS promotes lipid peroxidation and can cause cell damage^57^. Regulating the dysfunction of OS which is related to the dysfunction of phospholipids is supposed to be a novel approach for cancer treatment^20,58,59^.

Given the occurrence of the metabolism disorder in tumor cells, the metabolite alteration in PC samples was explored. After taking an intersection of the different metabolites between tumor samples and normal samples and different metabolites between tumor samples and para-carcinoma samples, the key metabolites differentially generated in PC were identified. Among all the metabolites, diacylglycerol, glycerophosphocholine, and glycerophosphoethanolamine were the main differentially-produced metabolites in PC samples. As a result, the disorder of PM was regarded as essential in the development of PC.

Due to the influence of OS on PM, the potential mechanisms of OSPM-related genes in PC were investigated. After differential expression analysis, univariate Cox regression analysis, and KM survival analysis, 21 prognostic OSPM-related DEGs of 3,197 OSPM-related genes emerged as OSPM function genes. Subsequently, ssGSEA was performed to assess the OSPM function of all samples, and then PC samples were divided into three clusters based on the ssGSEA function score of normal samples. Samples in cluster 1 (OSPM-active cluster), with relatively high ssGSEA function score, had a poor prognosis. In the in-depth exploration, the discrepancies of the expression of tumor suppressor genes and oncogenes (i.e., COL24A1, FAT2, CENPJ, ECT2, PXDN, CCND1, ADAMTS12, and KMT2C) and ICGs (HAVCR2, TNFSF4, TNFRSF9, PDCD1LG2, CD80, TNFSF9, ICOS, CD70, IDO1, PDCD1, and HHLA2) may result in different prognoses.

In addition, the 21 genes were summarized in pan-cancer. Compared with their corresponding normal samples, these genes were differentially expressed in tumor samples and were related with their prognosis. The high frequencies of CNV and SNV and the methylation level of promoters might be related to the alteration of the gene expression levels. The above findings shed new light on the in-depth research concerning OSPM in cancer.

In view of the impact of 21 prognostic OSPM-related DEGs on PC patients, an OSPM-related prognostic signature was developed utilizing the most appropriate variables selected from the 21 genes. Eight genes (i.e., SLC2A1, MMP14, TOP2A, MBOAT2, ANLN, ECT2, SLC22A3, and FGD6) were included in the signature. Interestingly, all the eight genes had increased expression levels in the high-risk samples, and the protein levels of SLC2A1, MMP14, TOP2A, ANLN, and SLC22A3 also increased in tumor samples compared with normal samples. Additionally, the expressions of ECT2, FGD6, MMP14, and SLC2A1 were related to the staging of the tumor. The potential roles of these eight genes in PC have been identified in other studies, and all these genes were demonstrated to combine with unfavorable functions in pancreatic cancer. Solute carrier family 2 member 1 (SLC2A1), known as glucose transporter 1 (GLUT1), is reported to express highly in many cancers and promote cancer growth^60–62^. It is reported to be highly enriched in active glucose metabolism subtype, which is associated with lower survival rate^63^. Matrix metalloproteinase 14 (MMP14) was also identified as a prognostic gene in PC^64^. Certainly, knock-down of MMP14 was proven to prevent the invasion of pancreatic cancer^65^. DNA topoisomerase 2-alpha (TOP2A), as the downstream molecule of DGCR5, could influence the sensitivity of pancreatic cancer cells to gemcitabine^66^. Membrane-bound O-acyltransferase domain-containing 2 (MBOAT2), correlated with decreasing infiltration of CD8 + T cells and KRAS activation, has been demonstrated as an unfavorable biomarker in pancreatic cancer^67^. Anillin (ANLN) promoted the progression of pancreatic cancer through inducing EZH2 up-regulation by mediating the miR-218-5p/LASP1 signaling^68^. Epithelial cell transforming 2 (ECT2) was regulated by Yes-associated protein 1 and mediates pancreatic cancer progression and metastasis^69^. Ephrin-2 was found to promote the migration and invasion of PC through targeting of miR-557^70^. Solute carrier family 22 member 3 (SLC22A3) showed up-regulation in pancreatic cancer when compared with non-neoplastic pancreatic tissues, and patients with high expression of SLC22A3 may benefit from nucleoside analogs^71^. Faciogenital dysplasia 6 (FGD6) was found to promote the proliferation, macropinocytosis, and tumor growth both in vitro and in vivo in pancreatic cancer^72^. Our results suggested that the expression of MBOAT2 mainly existed in the epithelium, and the expression of MMP14 primarily existed in fibroblasts, while the expression of FGD6 mainly existed in progenitor.

Based on the OSPM-related signature, PC samples were divided into high- and low-risk subgroups. Further investigation of the discrepancies between these subgroups indicated that the high-risk subgroup showed statistically different immune response compared with the low-risk subgroup. Immune system disorders in PC may result in the poor prognosis. Also, the expressions of ICGs (i.e., SLC2A1, FGD6, MMP14, MBOAT2, TOP2A, ANLN, SLC22A3, and CD70) were up-regulated in the high-risk subgroup in both the training cohort and the test cohort. All these discrepancies related the risk of PC samples were potential targets for the treatment of PC.

This study has a few limitations that need to be addressed. Our signature was developed according to the analyses of retrospective data. Although the expression levels of genes in the signature were explored at both transcriptional level and translational level according to the online databases, additional basic exploration is required for further demonstration. Additionally, large clinical cohorts are needed to confirm the accuracy of the signature.

## Conclusion

In this study, metabolomics analyses uncovered alterations in PM, while transcriptomic studies revealed differential expression of OSPM-related genes in PC. Then three OSPM-based PC subtypes were identified, and an OSPM-related signature was developed, which helped to unmask the OSPM-based discrepancies of prognosis, tumor microenvironment, immune response, and the expression of immune checkpoint genes in PC. Finally, the expression levels of genes in the signature were further demonstrated. All these findings are valuable for estimating prognosis and facilitating individualized treatment in pancreatic cancer.

### Supplementary Information

Supplementary Information.

## Data Availability

The metabonomic data used in the current study can be downloaded from the supplementary materials of Liu’s study (https://doi.org/10.3389/fonc.2022.991051). The genomics and transcriptomics data used in the current study can be downloaded from the TCGA (https://portal.gdc.cancer.gov/), GEO (https://www.ncbi.nlm.nih.gov/geo/), ICGC (https://dcc.icgc.org/), ArrayExpress (https://www.ebi.ac.uk/biostudies/arrayexpress) platforms.
