# Supplementary material for: Multi-omics analyses based on genes associated with oxidative stress and phospholipid metabolism revealed the intrinsic molecular characteristics of pancreatic cancer
Source: Sci Rep. 2023 Aug 21;13:13564. doi: 10.1038/s41598-023-40560-4 (PMC10442332; doi:10.1038/s41598-023-40560-4)
Supplement: Supplementary file 1 — Supplementary Information. [file 41598_2023_40560_MOESM1_ESM.pdf]

Supplementary materials

Multi-omics analyses based on genes associated with oxidative stress and phospholipid metabolism revealed the intrinsic molecular characteristics of pancreatic cancer

Hongdong Wang[1], Hui Guo[2], Jiaao Sun[3], Yuefeng Wang[1]*

[1]Department of Hepatobiliary Pancreatic Surgery, The Second Affiliated Hospital of Dalian Medical University, Dalian, China;

[2] Department of General Surgery, The First Affiliated Hospital of Dalian Medical University, Dalian, China;

[3] Department of Urology, The First Affiliated Hospital of Dalian Medical University, Dalian, China

*Correspondence: Yuefeng Wang, wangyuefengdy2y@163.com, Department of Hepatobiliary Pancreatic Surgery, The Second Affiliated Hospital of Dalian Medical University, Dalian, China.

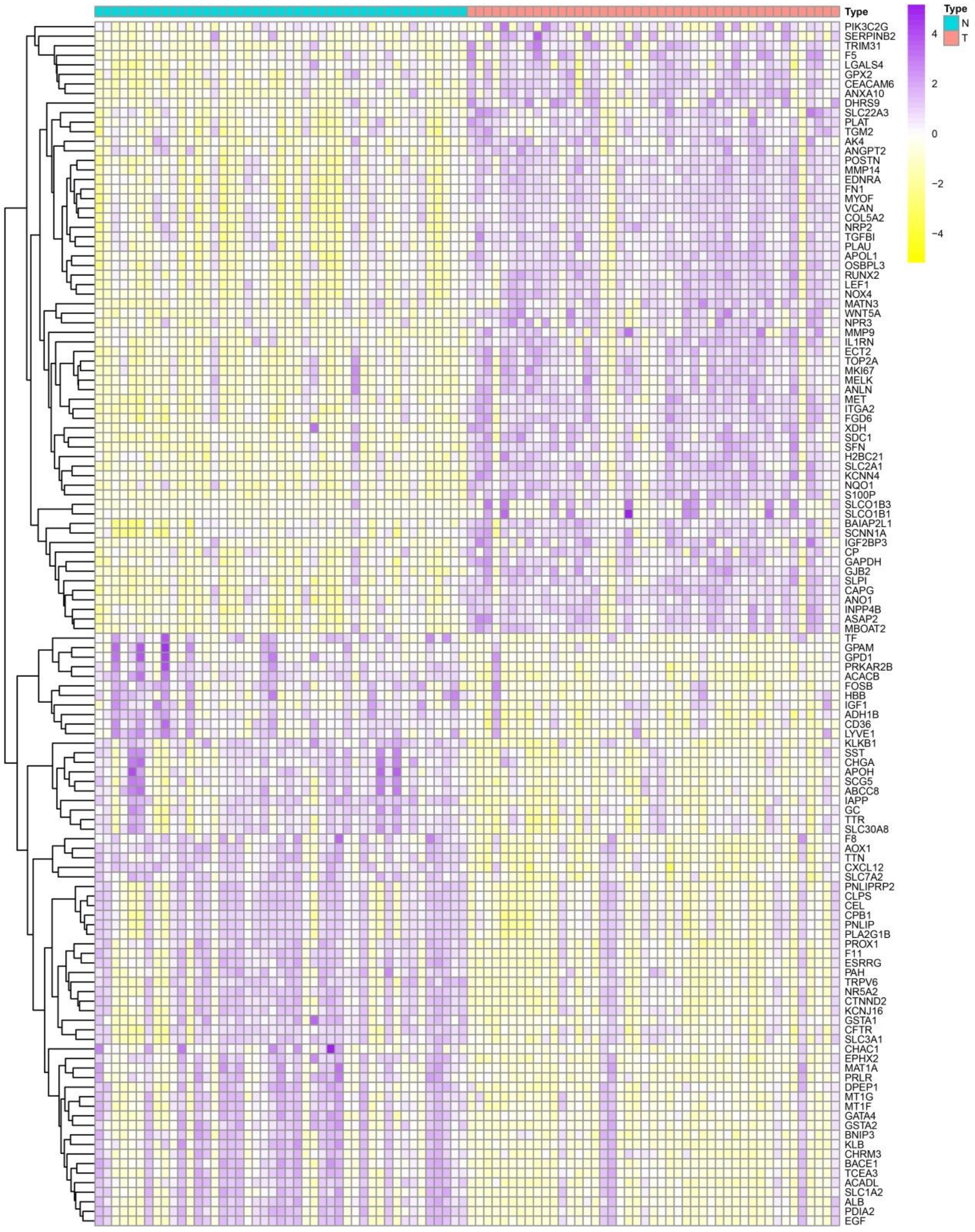

Supplementary Figure 1: The DEGs between tumor and normal samples analyzed in GSE28735.

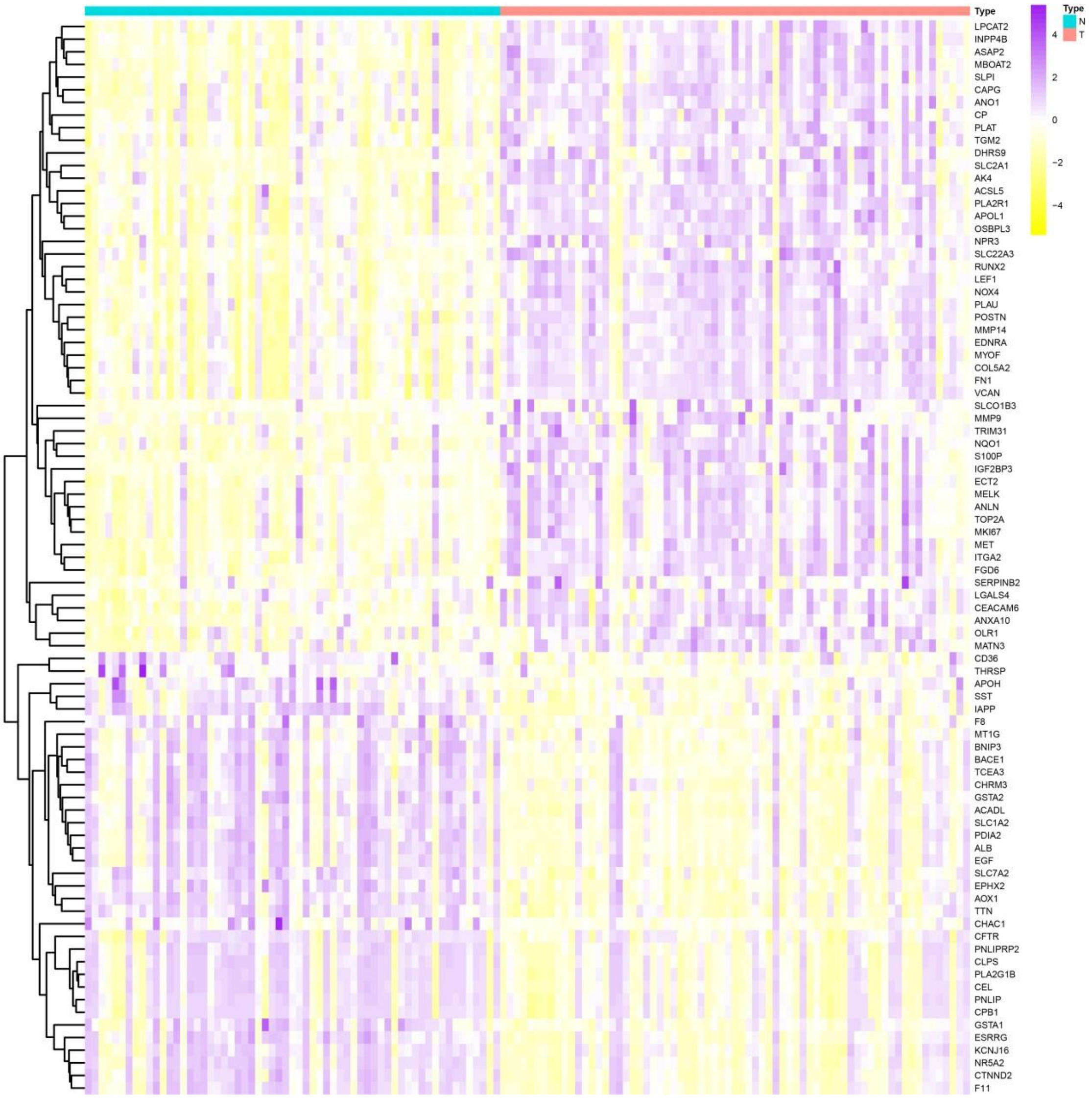

Supplementary Figure 2: The DEGs between tumor and normal samples analyzed in GSE62452.

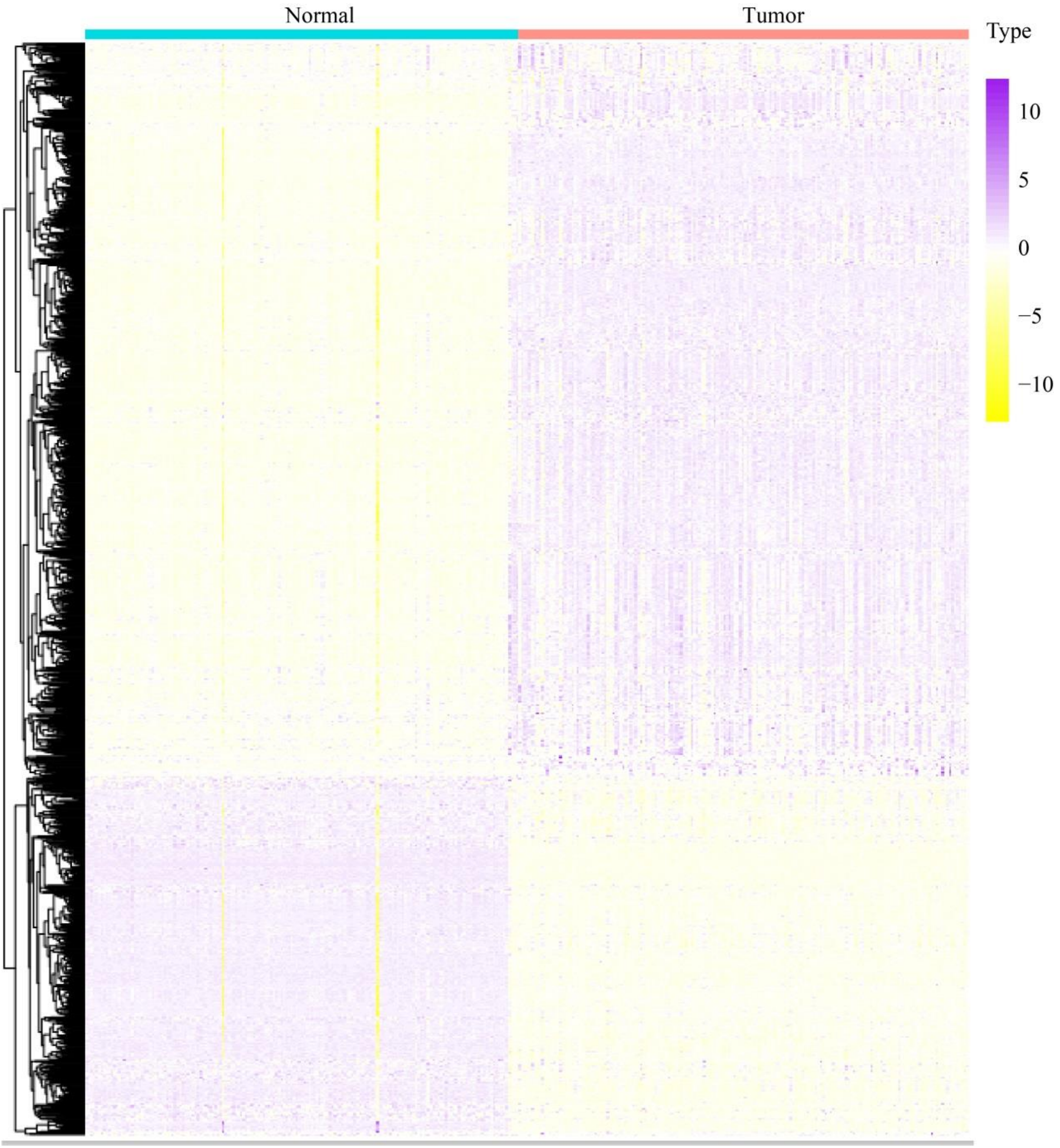

Supplementary Figure 3: The DEGs between tumor and normal samples analyzed in TCGA dataset combined with GTEx dataset.

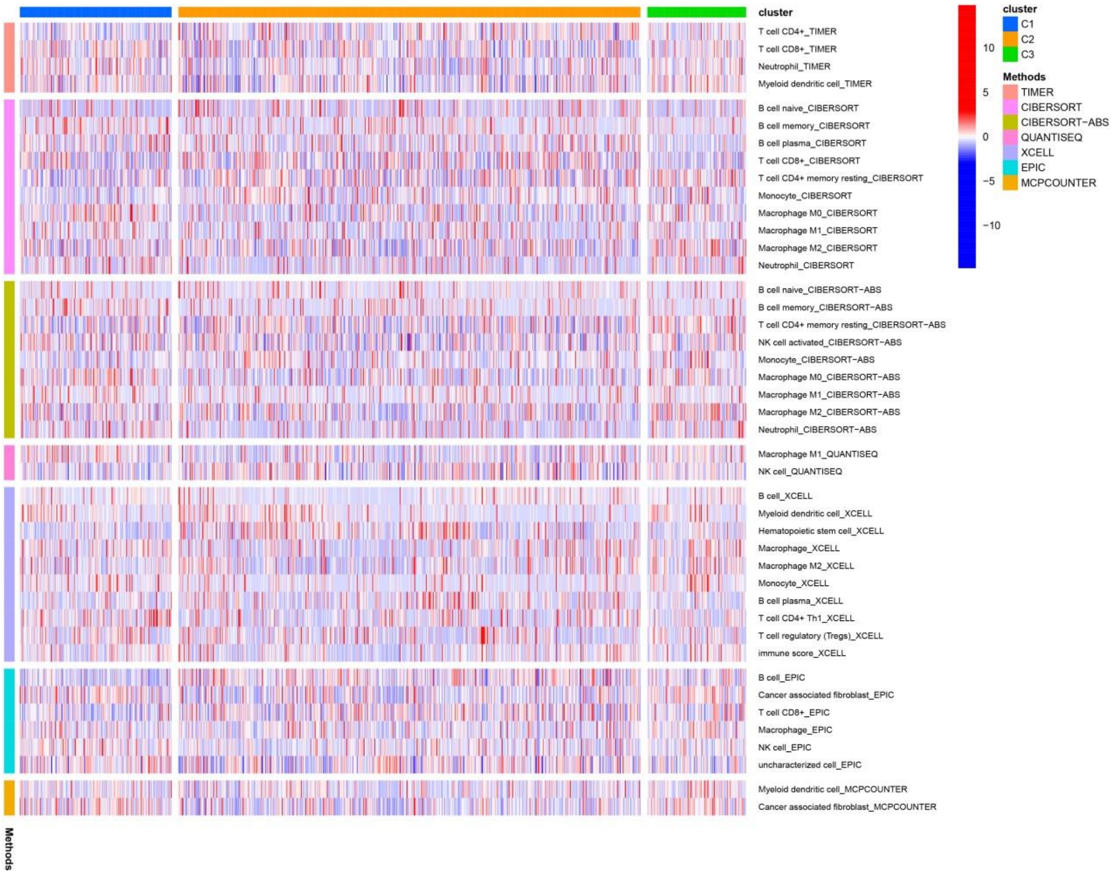

Supplementary Figure 4: The ICI in the three clusters.

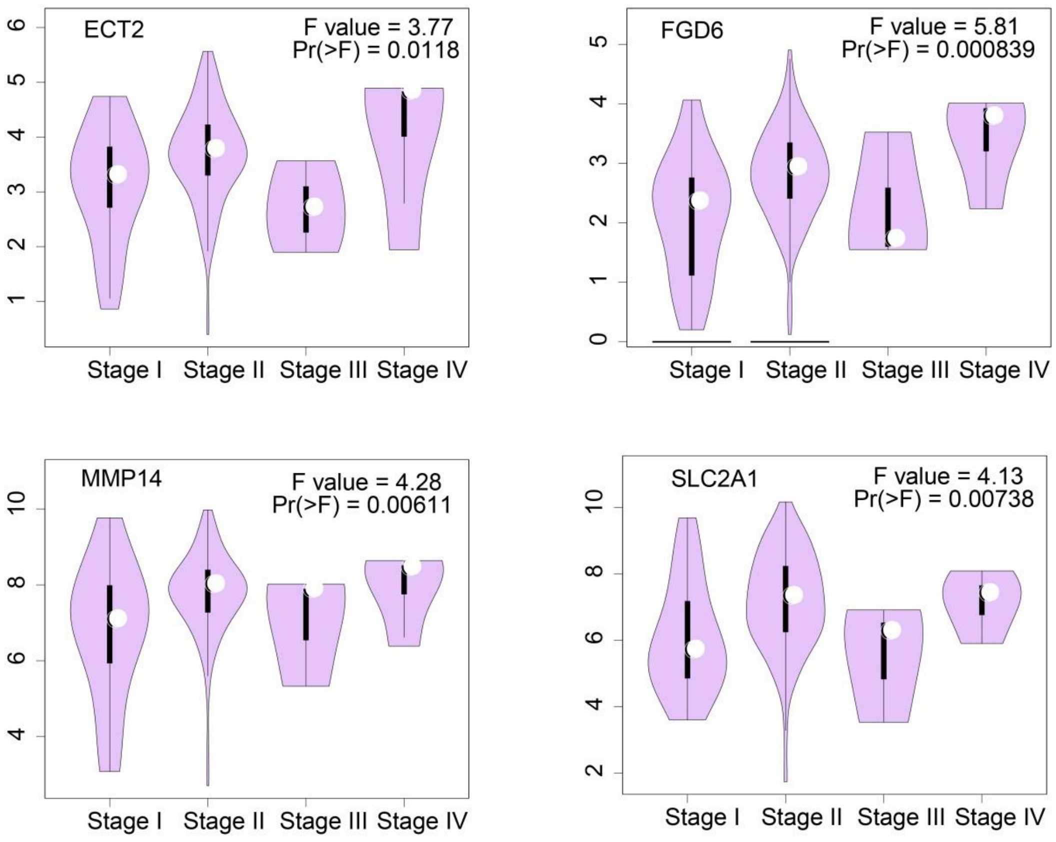

Supplementary Figure 5: The expression of the genes in the signature in different tumor stage.

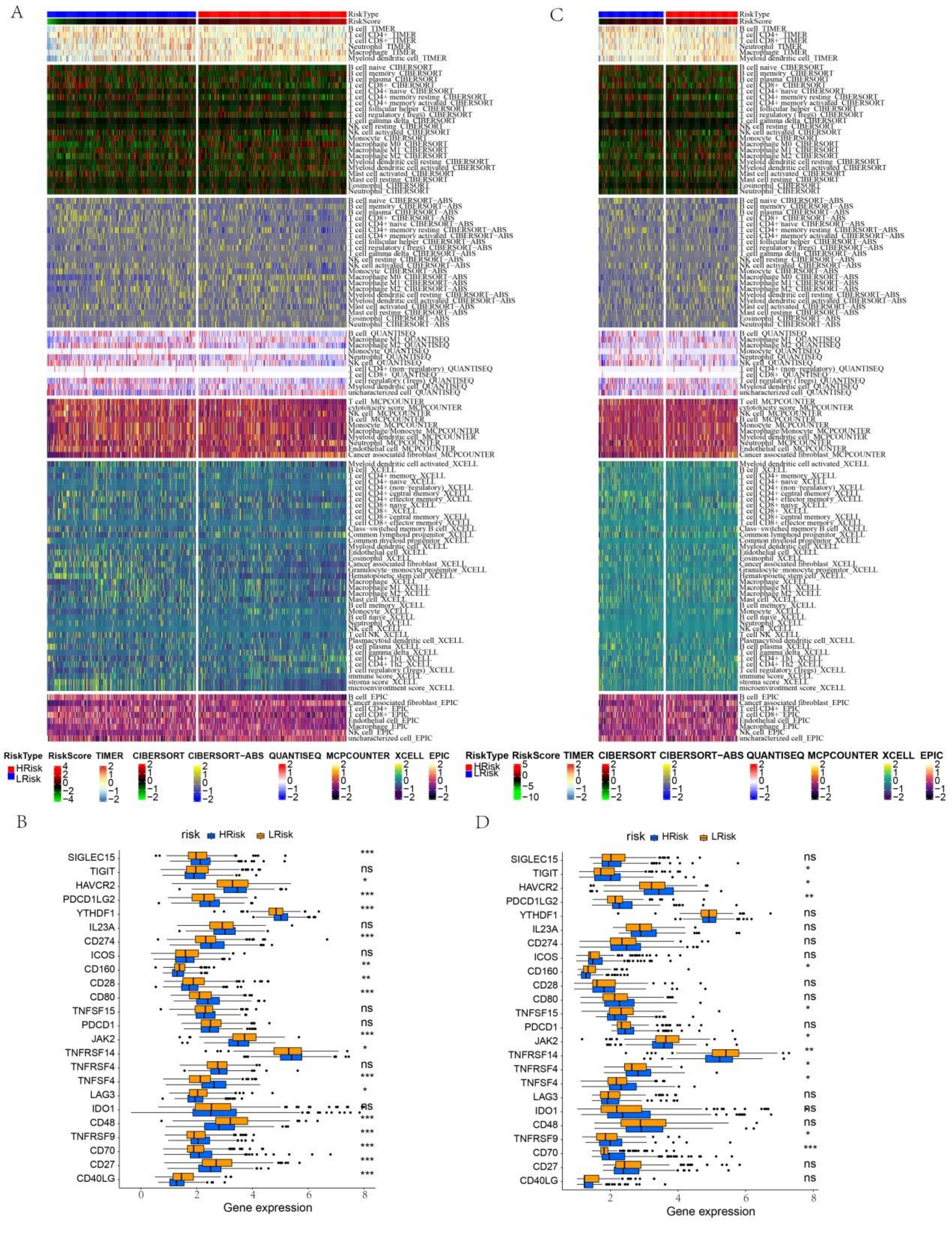

Supplementary Figure 6: The immune responses and the expression of ICGs in the training cohort (A, B) and the test cohort (C, D).

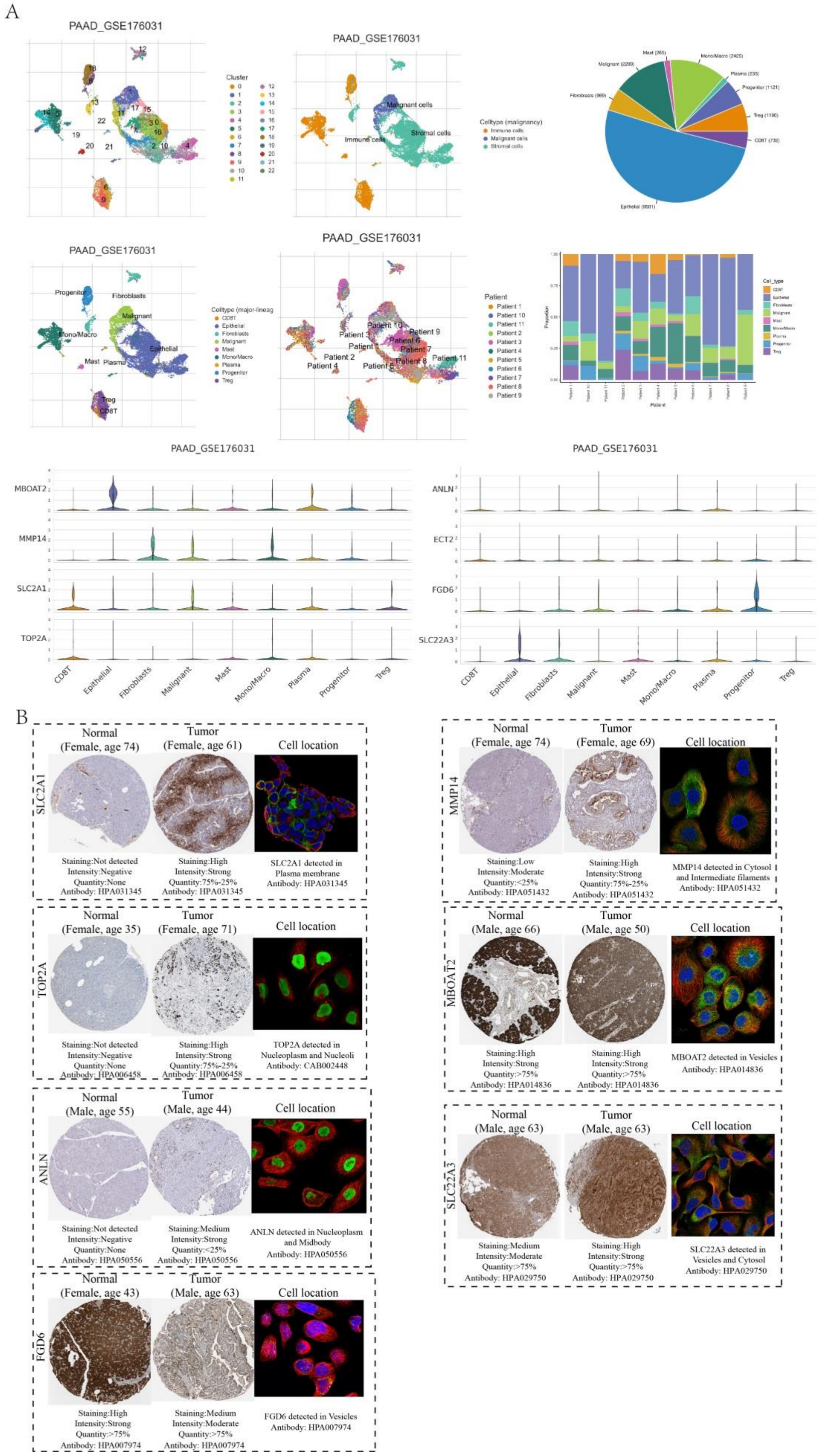

Supplementary Figure 7: Single-cell expression levels (A) and protein levels (B) of the genes in the OSPM-related signature.

Supplementary Table 1: Gene list of 72 intersected OSPM-related DEGs.
FN1
VCAN
CPB1
PNLIP
PLA2G1B
MT1G
CLPS
POSTN
MYOF
CEL
PNLIPRP2
LGALS4
PLAT
SLPI
ITGA2
MET
MMP14
COL5A2
BNIP3
PLAU
SLC2A1
CEACAM6
TGM2
APOL1
NR5A2
BACE1
INPP4B
CAPG
FGD6
OSBPL3
TCEA3
ASAP2
EDNRA
ANO1
AOX1
CD36
PDIA2
MBOAT2
ALB
AK4
NQO1
LEF1
EGF

ECT2
CTNND2
F11
NOX4
ANXA10
KCNJ16
TOP2A
S100P
SLC22A3
ACADL
MATN3
TTN
NPR3
RUNX2
MMP9
EPHX2
MKI67
GSTA1
MELK
F8
TRIM31
SERPINB2
ANLN
GSTA2
SLC1A2
DHRS9
CHRM3
ESRRG
SLCO1B3
